# Current Status and Potential of *Moringa oleifera* Leaf as an Alternative Protein Source for Animal Feeds

**DOI:** 10.3389/fvets.2020.00053

**Published:** 2020-02-26

**Authors:** Bin Su, Xiaoyang Chen

**Affiliations:** ^1^College of Forestry and Landscape Architecture, South China Agricultural University, Guangzhou, China; ^2^State Key Laboratory for Conservation and Utilization of Subtropical Agro-bioresources, Guangzhou, China; ^3^Guangdong Key Laboratory for Innovative Development and Utilization of Forest Plant Germplasm, Guangzhou, China

**Keywords:** *Moringa oleifera*, nutritional value, protein feedstuff, feeding effect, processing methods

## Abstract

The increased consumption of livestock, poultry, and fish products in people's diet threatens to drive production toward the use of more and more conventional crops in animal feeds. In this context, alleviating the tightening grain crop supply and ensuring the healthy development of animal husbandry through innovations in protein feedstuff production remain considerable challenges. *Moringa oleifera* is a miracle tree species with abundant nutrients, high protein biological value, and good feeding effect. As a new protein feedstuff, *M*. *oleifera* has great potential in alleviating the feeding crisis. Here, we review available literature regarding the characterization of *M*. *oleifera* in the field of animal husbandry in terms of nutrient content, digestion, and absorption characteristics, and feeding effects and present current challenges in using *M*. *oleifera* as animal feed.

## Introduction

The global demand for meat products worldwide has increased rapidly in recent years. The Food and Agriculture Organization of the United Nations (FAO) reported that the consumption of global meat products increased by 1.2% from 2017 and reached 336.4 million tons in 2018 (1) To sustain such high consumption, a parallel increase in the volume of animal feed production is inevitable. According to Oxfam's report, farm animals consume more than 30% of global grains, of which 90% is soybean; these grains are mainly consumed in factory farms (2). The ever increasing ruminant sector generates a significant amount of anthropogenic greenhouse gas emissions and thereby contribute to climate change. During ruminal fermentation, a digestive process in which microbes decompose and ferment food in the digestive tract or rumen, ruminants produce methane (CH_4_) and emit via belching. According to a FAO report, the livestock sector accounts for approximately 18% of CH_4_ and 9% of carbon dioxide (CO_2_) production (3). When social, economic, and environmental challenges are considered, conventional forages and feeding patterns are clearly unsustainable for animal husbandry. To achieve economically viable and environment-friendly forage production and reduce greenhouse gas emissions, researchers have exerted considerable effort to exploit agricultural by-products, tree foliage, and plant leaves to supply adequate nutrients and alter the feed composition. The use of tree leaves as feed ingredients is an effective mitigation strategy because tree leaves usually have higher nutritive value than grasses and thus more attractive to herbivores (4). Furthermore, tree leaves contain bioactive compounds, such as saponins and condensed tannins, which have antimicrobial properties that can be exploited in ruminant production to reduce CH_4_ emissions and improve fermentation efficiency.

**Figure 1:**
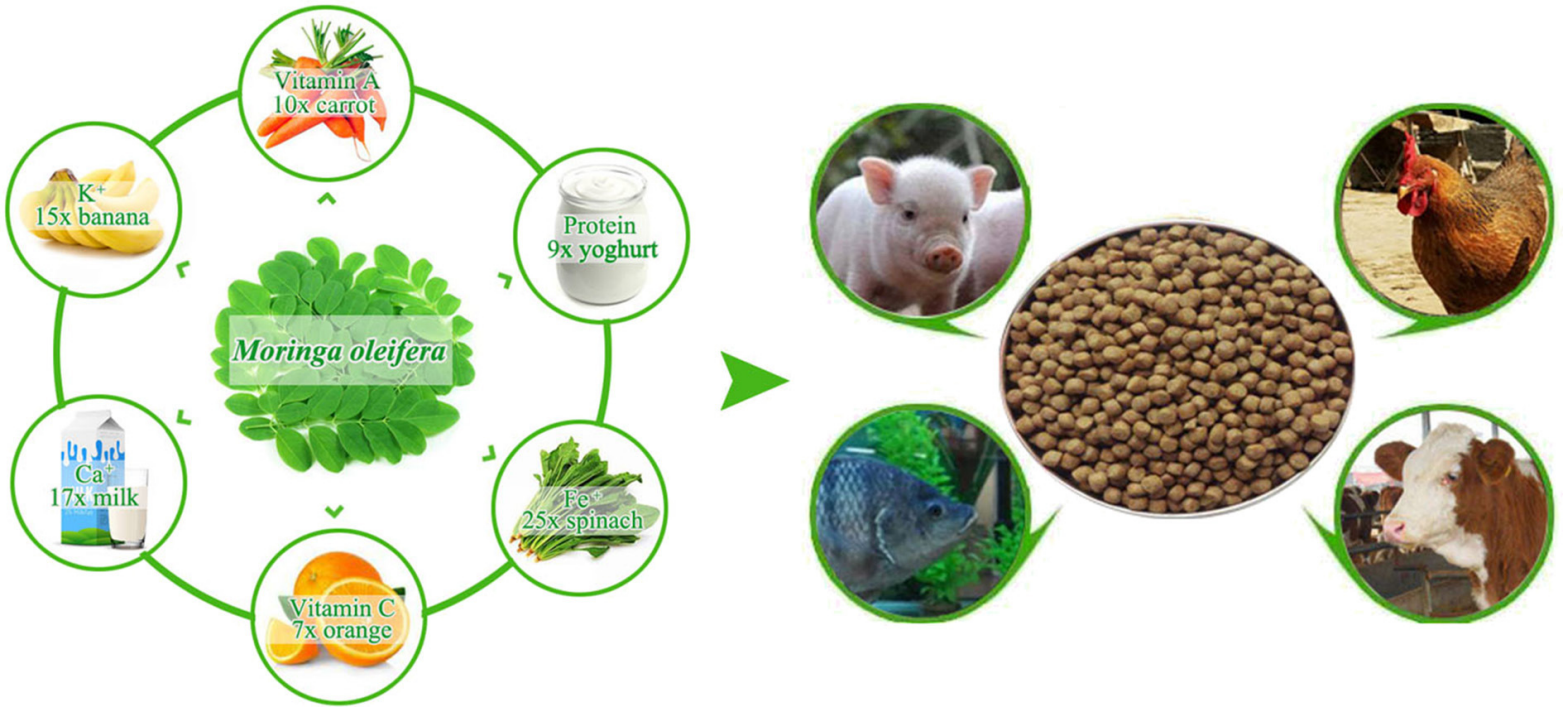
The nutritional characteristics of *Moringa oleifera* leaf and its potential use for feedstuffs.

*Moringa oleifera* is a rapidly growing soft wood plant that is mainly distributed in tropical and subtropical zones. In recent years, *M*. *oleifera* has increasingly attracted the attention of researchers in animal husbandry because of its comprehensive nutritional, antioxidative, and medicative attributes (5). *M*. *oleifera* leaf contains high amounts of crude protein, vitamin, mineral, and fatty acid. *M*. *oleifera* can provide 9 times more protein than yogurt, 17 times more calcium than milk, 7 times more vitamin C than oranges, 10 times more vitamin A than carrots, 25 times more iron than spinach, and 15 times more potassium than bananas [(6), Figure 1]. Given these excellent nutritional values, a daily intake of 10 g of *M. oleifera* leaf powder can help malnourished children to recover their body weights and enhance health indicators within a short time (7). Moreover, the consumption of *M*. *oleifera* leaf strengthens neural response, enhances immune functions, and improves health because of the large amounts of microelements and polyphenol antioxidants (8). Aside from promoting animal productivity and favorably influencing lipid composition, the potent antioxidant in *M*. *oleifera* leaf prevents meat products from deterioration (9–11). Although the relatively high content of indigestible substance in woody plants can affect the feed intake and digestion rate of livestock (12, 13), 1 kg of *M*. *oleifera* leaf contains only 12 g of tannins, which is lower than that in other woody plants (5). Studies have been carried out to evaluate the feeding effects of *M. oleifera* leaf meal on various animal species, including cattle (14), goats (10), chickens (15), and fish (12). All these studies concluded that *M. oleifera* leaf can be used as an alternative protein source for animal husbandry (Figure 1). Here, we characterized *M*. *oleifera* in terms of nutrient content, digestion and absorption characteristics, and feeding effects, and then presented current challenges in using *M*. *oleifera* in animal breeding.

## Botanical Characterization and Cultivation

*M. oleifera* is a slender softwood tree that belongs to the monogeneric family Moringaceae. It is a perennial plant with a height ranging from 5 to 12 m (16). Growing mostly at the tips of branches, tripinnate compound leaves are feathery with greenish elliptical leaflets that are 1–2 cm long (17). The flowers have yellow-white petals with light fragrance. The fruit is a trilobed capsule with a long slender pod morphology. During vegetative growth, the fruit pod is green and contains 15–20 seeds and changes to brown at maturity (18). The dry seeds are round or triangular, and each kernel is surrounded by a lightly wooded shell with three papery wings. *M. oleifera* grows in tropical and subtropical regions, especially in the areas with an average annual rainfall of 1,000–2,000 mm and high solar radiation (19). This plant, owing to its good drainage, can adapt to a wide range of soil types and has the best grow trend in slightly alkaline sandy loam soil (20). As an important economic crop, *M. oleifera* is now cultivated broadly in India, South China, and some parts of Africa (21).

*M. oleifera* yields a high amount of biomass ranging from 43 to 115 t/ha annually (22). With regard to leaf production, the leaf fresh weight yield of the plant is 1–5 kg per tree annually. This value is equivalent to 10,000–50,000 kg/ha annually at 1 m × 1 m spacing (23). In a 10 cm × 10 cm spacing, leaf yield is 7–8 kg/m^2^ at the first cutting in well-irrigated, drained, and fertilized beds, with up to seven cuttings a year (24). Although this intensive farming pattern can yield high amounts of leaves in a small area, it is unsuitable for large-scale plantation due to the large quantity of seeds required. A row spacing of 0.75 m × 1 m is the best cultivated pattern for leaf production in a large farming area (25). Biomass yields vary widely depending on farming conditions, including variety, soil type, climate, fertilization, and irrigation system. In practice, the best harvest can be obtained under warm and dry conditions and regular fertilizer supplement and irrigation (19).

## Nutrient Content of *M. oleifera*

*M. oleifera* as animal feed has attracted interest owing to its rich nutrients and low antinutrient content. Extensive literature on the analysis of nutrient composition showed that an *M*. *oleifera* leaf contains great amounts of proteins, minerals, vitamins, and other secondary metabolites. On a dry matter basis, the crude protein content of an *M*. *oleifera* leaf ranges from 23.0 to 30.3% (24), which is higher than the contents in *Medicago sativa* and *Morus alba* and approximately twice the contents of *Lespedeza bicolor* and *Caragana korshinskii* [(26), Table 1]. The total content of crude fiber of *M. oleifera* leaf is as low as 5.9% (24). Compared with soybean meal, which is considered as a standard feedstuff, their fiber contents were almost equivalent. In general, a low content of crude fiber indicates good palatability for animals. Another striking feature of *M*. *oleifera* leaf is its high mineral content, with ash content of up to 12.0%, which is significantly higher than that in soybean or corn meal (24, 27). Moreover, *M. oleifera* leaf contains ~7.09% lipids (27), and this amount is higher than the amounts in other woody plant forages. Notably, more than half (57%) of fatty acids in an *M. oleifera* leaf are unsaturated fatty acids, among which α-linolenic acid has the highest content [44.57%, (28)]. Unsaturated fats are the important components of human diet because they can decompose and esterify cholesterol, retain normal cholesterol level, and thereby reduce the risk of atherosclerosis and other cerebrovascular diseases.

**Table 1:** Comparison of nutrient contents (%) in *M. oleifera* leaf and other forages.

**Feedstuff Category**	**Plant species**	**Crude protein**	**Crude lipid**	**Crude fiber**	**Ash**
Woody Plant forages	*Moringa oleifera*	23.0–30.3	7.09	5.9	7.6–12
	*Morus alba*	21.2–29.8	5.5	6.9	11.6
	*Broussonetia papyrifera*	21.0	3.2	9.1	12.1
	*Caragana korshinskii*	9.9	3.2	34.4	6.7
Conventional Crop forages	Alfalfa meal	19.1	2.3	22.7	7.6
	Soybean meal	25.5	17.3	4.3	4.2
	Corn meal	9.4	3.1	1.2	1.2

*The data were calculated based on dry matter*.

Conventionally, amino acids, as nutrients, occupy the dominant position in the nutritional index of feedstuff. The total content of amino acids in feeds and whether amino acid composition meets a feeding target can directly affect feeding effect (29). *M*. *oleifera*, given its amino acid content, can be used as the main amino acid supplement for animal food in combination with other conventional forages. Phytochemical analyses showed that *M*. *oleifera* contains 16–19 amino acids, including the 10 essential amino acids, namely, threonine, tyrosine, methionine, valine, phenylalanine, isoleucine, leucine, histidine, lysine, and tryptophan (28, 30). *M. oleifera* has higher lysine, leucine, histidine, glutamic acid, valine, isoleucine, alanine, phenylalanine, and arginine contents, which is significant than that in other woody plants [(26, 31), Table 2]. Animals cannot synthesize some amino acids, or the synthesis rates and quantities of some amino acids in animals cannot meet the requirements for animal growth. Thus, some amino acids must be artificially supplied in animal feed (32). Amino acids that cannot be synthesized by animals are called essential amino acids. For the synthesis of a specific protein, the required essential and non-essential amino acids must be present at the site of synthesis according to the requirement of an animal. Otherwise, shortage in an amino acid may limit the use of other amino acids in the diet. The first amino acid to limit protein synthesis is termed as the first limiting amino acid. When the insufficient content of this amino acid is resolved, the next amino acid that limits synthesis is the second limiting amino acid (31). The first limiting amino acid in soybean meal is methionine, while that in corn meal is lysine (33). Therefore, the balance between essential and non-essential amino acids in feed formulation should be considered. As shown in Table 2, the comprehensive pattern of essential amino acids in *M. oleifera* leaf is modest and accounts for 52.19% of total amino acids. Notably, *M. oleifer* has higher lysine content than *Broussonetia papyrifera* and *Caragana korshinskii* and has almost eight times the lysine content of corn meal. By contrast, the content of methionine in *M. oleifera* leaf is higher than that in alfalfa meal and is very close to that of corn meal but is only two-thirds that of soybean meal (Table 2). Inadequate amounts of sulfur amino acids can seriously affect enzyme synthesis (34). In practice, amino acid deficiency can be addressed by adding soybean meal or fish meal as supplement; this method not only increases the total amount of amino acids but also enhances forage palatability (26). Another sulfur-containing amino acid cystine should be appropriately supplemented into *M. oleifera* leaf meal so that the demand for sulfur-containing amino acids is met according to the requirement of animals.

**Table 2:** Comparison of amino acid contents (%) in *M. oleifera* leaf and other forages.

**Amino acid**	**Woody plant forage**				**Conventional crop forage**		
	***Moringa oleifera***	***Morus alba***	***Broussonetia papyrifera***	***Caragana korshinskii***	**Alfalfa meal**	**Soybean meal**	**Corn meal**
Lysine	1.64	1.80	1.25	1.24	0.82	2.43	0.22
Leucine	1.96	1.35	1.69	1.30	1.20	2.75	/
Isoleucine	1.18	1.43	0.89	0.78	0.68	1.57	0.26
Methionine	0.41	0.52	0.36	0.18	0.21	0.60	0.43
Phenylalanine	1.64	1.94	1.24	0.84	0.82	1.79	0.31
Tryptophan	0.49	0.27	0.32	0.30	0.43	0.64	1.03
Valine	1.41	1.76	1.40	0.99	0.91	1.70	0.26
Histidine	0.72	0.69	0.42	0.47	0.39	1.10	0.23
Threonine	1.36	1.31	0.91	0.75	0.74	1.44	0.40
Cystine	0.01	0.30	0.30	0.11	0.22	0.62	0.34
Tyrosine	2.65	0.82	0.32	0.69	0.58	1.53	0.08
Arginine	1.78	1.80	1.00	1.14	0.78	2.53	0.38
Serine	1.09	1.22	0.90	0.80	/	/	/
Glutamic acid	2.53	3.33	2.03	1.88	/	/	/
Aspartic acid	1.43	3.06	1.88	2.03	/	/	/
Proline	1.20	1.31	1.18	1.12	/	/	/
Glycine	1.53	1.57	1.06	0.77	/	/	/
Alanine	3.03	1.54	1.13	/	/	/	/

*The data were calculated based on dry matter*.

In general, the amount of ash represents the content level of total minerals. *M. oleifera* leaf is rich in mineral elements, such as calcium, iron, potassium, phosphorous, and zinc, which are key elements for animal growth and development. For example, *M. oleifera* leaf contains 24,700 mg kg^−1^ calcium, 4,400 mg kg^−1^ phosphorous, 318.81 mg kg^−1^ iron, 190 mg kg^−1^ magnesium, and 22.05 mg kg^−1^ zinc on a dry matter basis (27). The abundances of these minerals in *M. oleifera* leaf are relatively high compared with those in other tree leaves (31). In organisms, calcium is a necessary element for the formation of bones, teeth, and eggshell, the maintenance of nervous function, the reduction in capillary permeability, and the regulation of metabolism and enzyme activation (28). *M. oleifera* leaf powder can partially replace milk for children owing to its abundant calcium content (6). Interestingly, iron deficiency is a common characteristic in many plant-based foods, except those based in *M. oleifera* leaf. The amount iron provided by an *M. oleifera* leaf is 25 times that provided by spinach (27, 31). Moreover, although iron is abundant in a spinach leaf, its absorption rate is relatively low; the absorption level of iron in *M. oleifera* leaf is higher than that in spinach and other leafy vegetables (31). Iron is a key component of various proteins and participates in various biochemical reactions in the body. Iron deficiency affects the body's physiological functions (35). An appropriate amount of iron ions promotes animal growth, especially in the presence of zinc. High zinc content is another excellent characteristic of *M. oleifera*. Zinc, a necessary component of enzymes, plays an important role in tissue respiration and the metabolism of proteins, fats, sugars, and nucleic acids (36). An *M. oleifera* leaf contains a high amount of magnesium, which has positive effects on milk yield. Usually, determining the appropriate amount of magnesium in feedstuff for cows according to their growth stages is difficult due to their different requirements. However, given that cows have a flexible balance mechanism to handle excess magnesium ions, the addition of excess magnesium salt into their feeds is recommended (37).

Nowadays, plant-derived antioxidants are widely used as feed additives in husbandry. Animals consuming plant-based feed supplements with antioxidants show a strong antioxidation ability (38). The active ingredients of natural antioxidants are mainly polyphenols, polysaccharides, alkaloids, and vitamins (39). Generally, woody plants contain more secondary metabolites than herbaceous plants. *M. oleifera* leaves have abundant vitamins, flavonoids, phenols, and carotenoids. (40) compared the total contents of phenols and flavonoids of *M. oleifera* with those of cabbage, spinach, broccoli, cauliflower, and pea and found that the amounts of these compounds in *M. oleifera* leaves were twice those of household vegetables. *M. oleifera* leaves and leaf extracts have been used as feed additives in animal diets to improve meat quality owing to the leaves' abundant secondary metabolites (10, 41). In addition, the mean concentration of total carotenoids is 40,139 μg/100 g of fresh weight in *Moringa* leaf. Approximately 47.8% of this amount is β-carotene (42), which is one of the most important precursors for vitamin A, an active substance that promotes growth and reproduction and maintains various physiological functions, such as bone, epithelial tissue, and visual and mucosal epithelial secretion. Although vitamin A can be converted from β-carotene within an animal's body (28), vitamin-rich *Moringa* leaf can directly supply enough vitamin A for animal growth and development. In addition to vitamin A, *Moringa* leaves are also rich in vitamin E, which has antioxidant ability, and thus protect cells from harmful free radicals (i.e., reactive oxygen molecules) and help enhance cellular immunity. *M. oleifera* leaf supplements confer high antioxidant capacity on goats and broiler chickens, and this characteristic is reflected in meat oxidative stability and shelf life (10, 41). Given the above nutrient properties, *M. oleifera* is a nutritionally healthier feed than other vegetables.

## Antinutritional Factors in *M. oleifera* Leaves

Although woody plant leaves have high nutritional values, their application to animals' daily diets have some restrictions, such as the presence of excessive fiber and antinutritional factors, which can severely affect palatability and digestibility. Generally, the contents of antinutritional factors are vital indexes for determining whether a tree leaf is suitable as an animal feed. Antinutritional factors are defined as substances generated in natural feed ingredients through the normal metabolism of plants, which interact with the chemical composition or interfere with digestion or metabolic processes in the body by different mechanisms, and pose an effect contrary to optimum nutrition (43). Being an antinutritional factor is not an intrinsic characteristic of a compound but depends upon the digestive process of the ingesting animal (43). These factors can be divided into four types according to action principle: substances that inhibit protein digestion and utilization, substances that inhibit energy utilization, substances that increase the vitamin requirements of animals, and substances that disrupt the immune system. Of these substances, protease inhibitors, phytic acid, polyphenols, and plant lectins account for the vast majority and exhibit the main antinutritional effects on animals (31).

*M. oleifera* leaves are suitable for animal feed not only because of their great amounts of nutrients but also because they contain low amounts of antinutrients. Nevertheless, the antinutrient content in *M. oleifera* leaf varies depending on the genetic background of the plant, such as the cultivar and growing environment (44). For instance, the content of tannins present in *Moringa* leaves ranges from 12.0 to 20.6 mg g^−1^ (27, 45). Tannin is a phenolic compound that interacts with trypsin and amylase or with the substrates of these enzymes to form complexes that are not readily digestible, resulting in decreasing palatability and reducing feed intake. Drying, fermentation, and silaging treatments can reduce tannins by 15–30% compared with fresh foliage (46). Therefore, in practical applications, enhanced feeding effects may be achieved after these processes. Although an *M. oleifera* leaf contains saponins, which provides a bitter taste, their amounts in dry matter are only 4.7–5 g kg^−1^. This quantity cannot cause any adverse effects on livestock (28). Lignin is another harmful antinutrient commonly found in tree leaf forages. It is a key structural material for cell wall formation. A plant cell wall comprises an average of 23% lignin on a dry matter basis (47). Ruminants can use lignin as an energy source because rumen microbes can degrade lignin into monosaccharides. However, monogastric animals cannot digest lignin because their digestive tract is simple and thus cannot secrete related enzymes that degrade lignin (48). The adverse effects of lignin in *Moringa* leaf meal has not been studied, but *M. oleifera* leaves have relatively low fiber contents (5.9% in dry matter). At this percentage, adverse effects from lignin are negligible. Notably, lignin content in tree leaves tends to increase with growth time. Lignin accumulation in *M. oleifera* leaves can be prevented by shortening the cutting interval and harvesting young leaves during the early vegetative period. Moreover, phytates and oxalate contents in *M. oleifera* leaves are lower than those in other household vegetables (49). The content of phytates in an *M. oleifera* leaf is only 22.3 mg g^−1^ in dry matter (50). Similarly, oxalate content is 27.5 mg g^−1^, which is far lower than that in spinach leaf (125.7 mg g^−1^) or green amaranth leaf (100.5 mg g^−1^) (51). High concentrations of these antinutrients can severely affect the absorption of trace elements in food sources and hinder protein digestion.

## Dietary Application of *M. oleifera* Leaf in Animal Feeding

*Moringa* has a long history of use as a feedstuff. Feeding of *Moringa* seed was first reported in 1962 (17). Leaves (fresh leaves and dried leaves), young branches, and seed residues after oil extraction have been used to feed farm animals (24). In recent years, *Moringa* leaves have been widely used as substitutes for traditional protein feeds for monogastric animals (e.g., pig, rabbit, chicken), ruminants (e.g., cattle and sheep), and aquatic animals (24, 52, 53).

## Effects of *M. oleifera* Leaf On Non-ruminant Performance

Studies on the growth performance of broiler chickens demonstrated that *M. oleifera* leaf meal can significantly improve bowel health by balancing intestinal microflora, thus promoting weight gain (52, 54). Alabi et al. (55) verified that broilers fed with diets containing aqueous *M. oleifera* leaf extracts had increased body weights, low total feed intake, and improved feed conversion ratio compared with the control group. These results might be related to the presence of different bioactive components, which improve nutrient utilization in *Moringa* leaf extracts. In another study conducted by Onunkwo and George (56), there was no difference in the feed intake and body growth weight of broiler chickens when fed with soybean meal with *M. oleifera* leaf at the rate of 0.0, 5.0, 7.5, and 10%, demonstrating that *M. oleifera* leaf meal can replace partial protein source in poultry diets without causing any deleterious effects on growth performance. Rao et al. (57) studied the benefits of dietary supplementation with *M. oleifera* leaf meal on the performance, carcass attributes, immune, and antioxidant responses in 1–42-day-old commercial broiler chickens. They found that supplementation with *M. oleifera* leaf meal (500 and 1,000 mg/kg) improved humoral immune response in 42-day-old commercial broiler chickens and reduced lipid peroxidation in the liver without showing any negative effects on performance and carcass attributes. Similar results were also obtained by Ayssiwede et al. (58), who showed that *M. oleifera* leaf inclusion in groundnut cake meal of up to 24% did not cause any adverse effects on average daily gain, feed conversion ratio, total weight gain, mortality, carcass, and organs characteristics in Senegal chickens. Among all dietary treatments, 8 and 16% *M. oleifera* leaf in the diets were the most economically profitable formulas, which significantly increased the growth rate of chickens. Gadzirayi et al. (59) investigated the effects of *M. oleifera* leaf as a protein source substitute for soybean meal in poultry feeding. Although the feed intake under different treatments was not significantly different, the feed conversion ratio significantly differed as evidenced by the variation in weight gain. Hematological characteristics provide useful information for monitoring body health status. Broiler chickens fed with *M. oleifera* leaf meal had increased red blood cell counts, especially the groups supplied with 0.6% *M. oleifera* leaf powder in basal diet; the highest mean value of red blood cell count was recorded in these chickens (60). This result is not surprising because *M. oleifera* leaf is rich in iron ions, which are key components for hemoglobin and myoglobin formation. Given that the blood biochemical index is the important criteria for evaluating the quality and applicability of feeds, *Moringa* leaf meets the formulation standard for broiler feed.

The application of *M. oleifera* leaf meal in layer diets to improve laying performance and egg quality has been reported. In terms of laying performance, the addition of 5 and 10% *M. oleifera* leaves into cassava-chip-based diets for commercial egg strain chickens does not affect egg production (61). Likewise, replacement of 15 or 20% sunflower seed meal with *M. oleifera* leaf powder can significantly increase egg weight (62). Meanwhile, Abou-Elezz et al. (63) yielded a negative result; that is, they detected decreased linear relation between the *M. oleifera* leaf dosage and egg laying rate. This result may be due to the type of basal diets and the age of the harvested *M. oleifera* leaves. As age increases, crude fiber content increases, and this increase may affect feed intake. Egg nutrient composition is the main factor that consumers mostly consider. When *Moringa* leaf powder is used as a substitute for partial basal diet, egg nutrition improved, and egg yolk cholesterol level is reduced (64). Moreover, the presence of *Moringa* leaf increases egg high-density lipoprotein content while lowering cholesterol and low-density lipoprotein content (65). This nutrient change may be due to the presence of flavonoids in *Moringa* leaves; these flavonoids regulate the activity of follicular cell receptor and thereby reduces intracellular cholesterol content (66). Hence, a low proportion of *Moringa* leaf in basal diet may improve growth performance and egg quality.

Pork is a nutritionally complete food and is the most widely consumed meat around the world. Owing to growing concern about food quality, the pig farming industry has sought methods to improve pork quality and reduce feed cost. *M. oleifera* leaf meal is effective in improving pork quality. According to 64, finisher pigs fed with meal containing up to 5% *M. oleifera* leaf showed no negative effects on feed conversion ratio and carcass traits (e.g., cutability and backfat thickness) and even exhibited a strong acceptable odor and a striking dark red color in meat after prolonged refrigerated storage. Surprisingly, although the pigs that received diet containing more than 5% *M. oleifera* leaf meal showed increased daily feed intake, their feed conversion ratio was significantly poorer than the feed conversion ratio of trial groups fed with 0, 2.5, and 5% *M. oleifera* leaf meal (67). However, these results are contrary to the finding of Dany et al. (68), who reported that the inclusion of up to 40% *M. oleifera* leaf in Mexican hairless pig feed did not affect growth performance. The increasing consumption of *M. oleifera* leaf meal resulted in an increase in the amounts of unsaturated fatty acids in subcutaneous fat and meat, which contribute to the consumer health. Commercial prestarter and starter pigs fed with 10% *M. oleifera* leaf did not show any difference in average daily gains (69). Hence, a specific *M. oleifera* leaf meal diet should be formulated for pigs according to their growth stages.

## Effects of *M. oleifera* Leaf On Aquatic Animal Performance

The use of *M. oleifera* leaf is also effective in aquaculture. Fermin and Reyes (70) reported that abalones fed with 13% *M. oleifera* leaf tend to grow faster and accumulate higher level of carcass protein and lipid than the control feed group. Given that *M. oleifera* is a locally available year-round in the Philippines, the use of *M. oliefera* leaves as alternative daily food for farmed abalones has been proposed. Adeshina et al. (71) used *Moringa* leaf meal for *Cyprinus carpio* and found that supplemented diets can enhance growth at the juvenile period. In addition, *Moringa* leaf meal significantly improve fish immune response. The authors finally stated that *M. oliefera* leaf meal can be used to replace 30% soybean in the diet of *C. carpio* juveniles. Similar results were obtained by El-Gawad et al. (72), who reported significant increase in white blood cell count and non-significant change in red blood cell count and hemoglobin levels in Nile tilapia fed with 5% *M. oliefera* leaf powder. Furthermore, *Aeromonas hydrophila* infection trial showed that all the fish samples in the experimental groups survived compared with only 20% of the controls. These results indicate that *M. oleifera* leaf powder-supplemented diets can enhance immunity and control disease infection caused by pathogens in Nile tilapia fry. Mansour et al. (73) evaluated the effects of increasing dietary levels of *M. oliefera* leaf on the growth and immune status of seabream. In addition to the high growth performance, they detected the upregulation of intestinal mucosal immunity genes in fish samples fed with 5 and 10% *M. oliefera* leaves. However, increasing the inclusion level of *M. oliefera* leaf to up to 15% significantly decreased the growth performance and feed utilization. Finally, the authors stated that *M. oleifera* leaf should be used within the 10% level in seabream diet. Richter et al. (74) also found that high proportion of *M. oliefera* leaves in feed (20 and 30%) depressed the growth rate of Nile tilapia. These results may be due to the existence of antinutrients, which could severely interfere with the absorption of nutrients in the feed. Therefore, feasible methods should be further explored to reduce the amounts of antinutrients before *M. oliefera* leaf meal is provided to fish. Progress has been made on processing methods for removing antinutrients. Makkar and Becker (75) used ethanol to process *M. oleifera* leaves; tannins, lectins, trypsin inhibitors, and saponins were not detected after processing, and phytate content was only 2.5%. In addition, after analyzing the amino acid pattern of the processed *M. oleifera* leaves, they found that the concentrations of all essential amino acids, including sulfur-containing amino acids, were higher than the concentration recommended by FAO (76). Afuang et al. (12) developed a similar method but used methanol instead to treat *M. oleifera* leaves. They found that the inclusion of processed Moringa leaf meal of up to 33% resulted in no significant difference in growth performance compared with that in the fish meal group. These studies indicated that solvent extraction method improves the nutritional quality of *Moringa* leaves, thus extending dosage in feeds. However, caution should be taken when using chemical reagents to process food because chemical reagent residues pose potential health risks to animals.

## Effects of *M. oleifera* Leaves On Ruminant Performance

The global demand for meat products will continue to increase because of population growth. Ruminants play an important role in providing high-quality proteins that are essential to human diets, and they are important sources of greenhouse gas emissions. Methane emission from ruminant livestock is ~100 million tons annually (3). Significant increases in total CH_4_ emissions from ruminant livestock can be prevented by reducing the intensity of emissions. The composition of animal feed is a crucial factor in controlling the amounts of CH_4_ produced. Methane is produced by methanogenic microorganisms (belonging to the Archaea) in the guts of ruminant livestock. Relatively recent ruminant CH_4_ reduction strategies have included the introduction of CH_4_ inhibitors, biological and chemical, with animal feed, to kill off or at least reduce the activity of the methanogenic microorganisms in the gut (77). *M. oleifera* leaves are effective natural methanogen inhibitors and thus considered alternatives for critical antibiotic feed additives for alternating ruminal fermentation pathways (78). Dong et al. (79) examined the effects of dietary supplementation with *M. oleifera* leaves on the production performance and fecal methanogenic community in lactating cows and found that *M. oleifera* improved milk fat content and changed the composition and diversity of methanogens. The fecal archaeal community in the control treatment was predominated by *Methanobrevibacter*. When *M. oleifera* was added to the diet, the abundance of *Methanobrevibacter ruminantium* decreased, but *Methanosphaera* and *Methanosphaera* sp. increased in abundance compared with those in the control group. Hence, *M. oleifera* may reduce CH_4_ emissions by modifying the composition of rumen microbiomes. Soliva et al. (78) observed a 17% decrease in daily methane emission with the complete extraction of *M. oleifera* leaves diet compared with diets containing rapeseed meal or soybean meal. Another *in vitro* study also indicated that CH_4_ emissions can possibly be reduced by up to 50% by replacing soybean meal with *M. oleifera* leaf meal (80). Similarly, (81) conducted an *in vitro* experiment in which *M. oleifera* leaf extracts were used on dietary corn grain to evaluate the effect on ruminal fermentation and found that high concentrations of *M. oleifera* extract delay the initiation of CH_4_ production, thus decreasing CH_4_ and CO_2_ production and total biogas production. This result is in good agreement with the results obtained by Dey et al. (82), who were able to reduce methane level and increase the degradability of organic matter *in vitro* by wheat straw supplemented with *M. oleifera* leaves in buffalo diet. Such effects might be related to the existence of saponins or tannins in *Moringa* leaves. Pedraza-Hernández et al. (83) proposed a novel and interesting strategy using *M. oleifera* extracts and live yeast cultures (*Saccharomyces cerevisiae*) as feed supplements; the aim was to explore the sustainable mitigation of CH_4_ emissions from goats. This study showed that the combination of *M. oleifera* extract and *S. cerevisiae* in diet is highly effective against methane production, achieving low emission (11.7%) at 72 h of incubation. In addition, *M. oleifera* extract and *S. cerevisiae* interaction mitigated proportional CO_2_ production significantly at 8, 24, 48, and 72 h of incubation period. This finding partially supports the results of (84), who reported that growth performance and CH_4_ emission increases and decreases, respectively, due to the dose-dependent supplementation. Hence, *M. oleifera* leaves could be used as effective natural feed additive in ruminant diets, not only to reduce CH_4_ production but also to enhance the ruminal efficiency of dietary nutrient use.

Compared with monogastric animals, ruminants can digest cellulose, lignin, and other secondary metabolites owing to their rumen structures. Ruminants can indirectly digest plant food because of rumen microorganisms in their stomachs, where digestive enzymes break down macromolecular substances. Given this unique digestive system, ruminants can easily handle forages from tree, such as *M. oleifera*, quickly. Supplementation of 25, 50, 75, and 100% *M. oleifera* leaf meal in daily diets as substitutes for cotton seed meals for rams did not change body weight gain relative to the body weight gain in the control group (85). Similarly, the trial of feeding goats with *M. oleifera* leaves did not show obvious distinction in feed intake, ruminating rate, and digestibility compared with those in *Leucaena leucocephala*, which has been used in livestock feed productions for a long time (86). Aregheore (87) found that goats fed with fresh *M. oleifera* leaves at 20 and 50% as replacement for batiki grass had higher live-weight gain and higher digestibility of dry matter, crude protein, neutral detergent fiber, and organic matter than the control group. Fadiyimu et al. (88) reported a predominantly high trend in crude protein intake, dry matter and nutrient digestibility, nitrogen retention, and hematological profile gain in West African dwarf sheep fed with *Panicum maximum* meal with 25% *M. oleifera* leaves supplements. According to (89), supplementation with *M. oleifera* leaf to replace 75% dry matter of berseem clover can improve feed utilization in Nubian goats. They also observed that *Moringa* diets increased serum total protein, albumin, and glucose levels but decreased cholesterol and triglyceride levels. This study demonstrated that dietary supplementation with *Moringa* leaves is a potential strategy to improve meat quality. Moreover, *M. oleifera* leaf extract is a powerful ingredient for increasing feed intake, and the digestibility of dry matter, organic matter, and neutral detergent fiber and does not affect the digestibility of crude protein in Nubian goats (90). As a nutrient source supplement to forage, *M. oleifera* leaf meal improves not only growth performance but also milk output and the quality of cows and goats (90–92). Kholif et al. (90) showed that dietary *M. oleifera* leaf extract (up to 20 ml dose in basal diet) can enhance milk yield by ~6% and energy-corrected milk yield by 12%. They also found that total saturated fatty acids in milk decreased by ~4.6–5.6%, whereas total unsaturated fatty acids increased by a ~11.5–13.9%. Babiker et al. (91) showed that the inclusion of 25% *M. oleifera* leaf powder as replacement for alfalfa hay in goat and ewe diets significantly affected milk composition, which had higher fat, lactose, and solid non-fat contents than the diet containing 40% alfalfa hay but lower cholesterol and glucose contents. Furthermore, milk energy contents and outputs also increased considerably in ewes and goats fed with *M. oleifera* leaves (91). Cohen-Zinder et al. (93) observed a similar rise in milk fat, energy, and yield in cows fed with a compounded meal with *M. oleifera* leaves, chopped wheat hay, and sugar cane molasses (at dry matter ratio of 370:540:90, respectively) compared with the control diet of wheat hay. This condition is probably due to the high contents of phenolic components with antioxidative activity in *M. oleifera* silage as a beneficial factor to rumen microbial population (94). Coincidentally, (95) found that cow diets with 20 and 40% *M. oleifera* resulted in 25 and 16% increase in daily milk yield and higher levels of nutrients in milk as compared with those in *Trifolium alexandrium* hay diet. In addition, (96) noted that *M. oleifera* leaf meal completely replaced maize silage in lactating dairy cows diet but did not affect dry matter intake, milk yield, or milk composition. However, cows fed with over 50% *M. oleifera* leaf meal showed low serum concentrations of total cholesterol, high-density lipoprotein cholesterol, and low-density lipoprotein cholesterol and higher serum concentrations of urea than the control group. Finally, the study suggested the use of at most 50% *M. oleifera* leaf meal to promote growth in lactating cows. Hence, *M. oleifera* leaves can be used as alternative protein sources for animal feeding, especially for ruminants.

## Processing and Quality Improvement of *M. oleifera* Leaf for Animal Feeds

The conventional method for processing woody plant leaves for animal feed involves drying and mincing fresh leaves. The processed leaves are then mixed with other commonly used forages in a control ration. This method is suitable for small batch production. Tree leaves contain relatively high antinutritional factors, which may have adverse effects on the growth performance of farm animals. To improve the palatability and reduce the content of antinutrients, different processing methods have been adopted to process *M. oleifera* leaves.

In principle, water boiling treatment can destroy most of antinutrients, such as protease inhibitors, plant lectins, and saponins. Sallau et al. (97) evaluated the effect of boiling, simmering, and blanching on the degradation of antinutrients in *M. oleifera* leaves and found a significant decrease in the contents of cyanide, oxalate, phytate, and trypsin inhibitor. This result is in agreement with that of a previous study, which indicated that heat can destroy some antinutrients and improve the digestibility and palatability of plant leaves for animals (98). Boiling is more effective in removing antinutrients than simmering and blanching. Similarly, drying can improve the nutritional quality of *M. oleifera* leaves. Mbah et al. (99) found that the amounts of antinutrients, such as phytate, oxalate, and saponin, significantly decreased after processing through different drying methods, including sun, shade, and oven drying). However, tannin content increases after drying. By contrast, (46) reported that sun and oven drying significantly decrease tannin content. Soaking treatment is another effective method for reducing the amount of antinutrients in tree leaves. Chanchay and Poosaran (100) reported that mimosine content can be reduced from 4.4 to 0.2%, and tannin content can be reduced from 37.6 to 0.3% in *Leucaena leucocephala* leaves by drying–soaking–drying treatments. This research provides a technical reference for the processing of *M. oleifera* leaves.

Biological treatment technology is a new emerging method and is highly effective in alleviating antinutrients and improving the nutrient bioavailability of tree leaf meals. Enzymolysis, fermentation, or their combination has been used in improving the nutritional value of *M. oleifera* leaves (31). In enzymolysis and fermentation, the action of enzyme preparation and microorganisms is desired (101). Exogenous enzymes have long been adopted in the feed industry to eliminate antinutritional factors, improve phosphorus absorptivity, and compensate for the deficiency of endogenous enzymes and promote animal growth performance. Upon ingestion, exogenous enzymes act on antinutritional factors present in plant-based feedstuff, such as phytic acid or non-starch polysaccharides (102). Given that each endogenous enzyme has its own substrate-specific activity, multiple enzyme preparation has become the preferred strategy and is widely applied in the feed industry (103). Some bacteria and fungi, including *Lactobacillus plantarum, Bacillus pumilusis, Bacillus subtilis, Aspergillus oryzae*, and *Saccharomyces cerevisiae* can produce complex enzymes during fermentation and improve the nutritional quality of food. For example, *L. plantarum* is used in the fermentation of corn pastes, sauerkraut, ogi, and other fermented food products of vegetable origin (104–106). This fungus can reduce the contents of antinutritional factors in food, such as phytates and fiber, owing to the production of phytase and β-galactosidase (107). In the food industry, *L. plantarum* is used as a starter culture to improve organoleptic properties, flavor, and texture, limit pathogenic bacteria growth, and increase the shelf life of foods (108).

Thierry et al. (109) showed that *M. oleifera* leaves fermented with *L. plantarum* can reduce the phytate content to 66.92% while increasing protein content and peptic digestibility to a value of 63.97%. Fermentation also increases iron availability in *M. oleifera* leaf powders. During fermentation, *B. pumilus* synthesizes endoglucanase to dissolve cellulose and release nutrients from plant cells. Solid fermentation with *B. pumilus* produces more soluble proteins than liquid fermentation (110). Wang et al. (111) proposed a similar strategy, in which *M. oleifera* leaves are fermented with *Aspergillus niger* as the fermentative microbe. They found that the amounts of free amino acids significantly increased during fermentation, and the essential amino acid pattern was much better than that of the FAO reference protein (111). Interestingly, the fermented leaves generated a sweet-smelling aroma, which may help to improve feed quality and palatability of *M. oleifera* leaves. Fermentation with *Rhizopus oligosporus* can produce complex enzymes, such as β-glucosidase, cellulase, and xylanase, which are essential to biotransformation. These enzymes can modify primary and secondary metabolites in plant leaves and enhance active compounds, such as polyphenols (112). The utilization of *R. oligosporus* in the fermentation of *M. oleifera* leaves increases protein content levels and alleviates the crude fiber (113).

In fact, it is not necessary to eliminate the antinutritional factors completely from *M. oleifera* leaves because low amounts of antinutritional factors, such as tannins and hydrolysable phenols which are antioxidants, in animal feed not only helps improve meat quality but also reduces methane emission from ruminants.

## Future Perspectives: the Way Forward

Although *M. oleifera* leaves have been widely applied to feed all types of animals, some challenges still need to be solved for large-scale feed production. The presence of endogenous antinutrients in plant leaf meals is one of the dominant limiting factors. Hence, high *M. oleifera* leaf inclusion levels in diets negatively influence animal growth performance. Tannins, phytic acid, and saponin are the main antinutrients present in *M. oleifera* leaves (31). Generally, antinutrients can decrease palatability, protein digestibility, and mineral bioavailability, and thereby limit the biological value and acceptance of *M. oleifera* leaf as a regular food source. Thus, the leaves should be appropriately treated before large-scale consumption. *M. oleifera* leaves should not be supplied to animals as raw materials and should be processed for the deactivation of antinutrients (31). Different strategies for limiting the negative effects of antinutrients and improving the nutritional quality of tree foliage are currently available. Physical, chemical, and biological methods can be employed to reduce or remove antinutrients; these methods include soaking, cooking, fermentation, selective extraction, irradiation, and enzymic treatment. Most physical methods, such as cooking and soaking, can significantly reduce phytate content in *M. oleifera* leaves (46, 99, 100). However, during treatment, certain nutritional elements, such as minerals, are lost. Hence, the replacement or the combination of this process with other techniques can be a good track for reducing antinutrients in *M. oleifera* leaves. Fermentation is by far the most effective and nutritionally beneficial process method, which renders food digestible, nutritious, and flavorful. In general, fermentation can reduce the levels of some antinutrients, particularly phytates, lignin, and cellulose, and poses a positive effect on the availability of iron and other minerals in leaves. In *M. oleifera*, fermentation reduces phytate content by 66.9% and increases digestible protein content (109, 111). Tannin, protease, and trypsin inhibitor contents are reduced by fermentation in other plant meals (114, 115). In comparison with soaking, the reduction effect of antinutrients obtained by fermentation is more significant. Hence, the use of fermentation for the treatment of *M. oleifera* leaves would be a good asset of the nutritional point.

*M. oleifera* is a successful crop in India, where a new cultivation management model and matching production line have been established for this tree. However, the cultivation of *M. oleifera* around the world is inadequate for stable and sufficient supply for food and feed industries. To the best of our knowledge, *M. oleifera* is mainly cultivated in the warm tropical parts of the world, especially in a few Southeast Asian countries; its cultivated area is relatively small, and its yields vary widely. Biomass yield is insufficient in limited areas mainly because of insufficient excellent cultivars. This condition constitutes an insurmountable obstacle to the expansion of *M. oleifera* cultivation around the world. *M. oleifera* is vulnerable to cold stress, especially in areas exposed to sudden cold wave and frequent or long winter frost (116). Poor resistance to cold stress is probably the critical factor impeding the northward expansion of this crop. Therefore, breeding new cultivars with stress tolerance characteristics may expand *Moringa* cultivation to a wide range of agroclimatic conditions.

For the simultaneous increase in abiotic stress resistance and yield potential, a suite of conventional and modern molecular breeding techniques is required. Fortunately, extensive research in India and China has provided valuable information on the genetic improvement of *M. oleifera* (117, 118). Genomic selection is a powerful and efficient breeding approach for improving complex and low heritability traits and enables the rapid selection of cultivars adapted to different climates and soils. In India, Horticultural College at Tamil Nadu Agricultural University is the earliest research center that focuses on *Moringa* breeding, especially in the aspects of germplasm collection and preservation and improvement of *Moringa* varieties. To date, 85 *Moringa* accessions, which mostly belong to *M. oleifera*, are available for commercial cultivation in different agroecological zones/across zones (119). These cultivars have different plant statures, branching and bearing habits, leaflet sizes and shapes, and pod lengths. Two *M. oleifera* cultivars, namely, PKM-1 and PKM-2, have been introduced in China, and their horticultural characteristics and adaptability under south China conditions have been evaluated. A series of field trials showed that PKM-1 and PKM-2 have high leaf biomass yields and are well-adapted to local climates. Their cultivated areas and production rates have steadily increased in the past few years (120). Another new genetic cultivar of *M. oleifera*, which was developed by researchers in Israel through 10 years of germplasm selection, exhibited good growth in drip irrigation systems at high planting densities [160,000 seeds/ha; (121)]. Under this planting pattern, leaf biomass of 35 t dry matter per hectare was obtained under four cuttings a year between July and November (93). Despite the progress made, ongoing research on second-generation traits (nutrition, flavor, and bioenergy) is slower than research on model plants with large breeding programs. By strengthening the genetic improvement program and developing mechanical mowing, silage fermentation, and appropriate agricultural policy, *M. oleifera* may become an important forage crop for animal husbandry.

## Author Contributions

BS wrote the manuscript. XC provided helpful comments on the article, and writing, review, and editing.

### Conflict of Interest

The authors declare that the research was conducted in the absence of any commercial or financial relationships that could be construed as a potential conflict of interest.
